# Supplementary material for: Are carbon emissions trading and green financial instruments synergistic? -Comprehensive quantitative research based on content analysis
Source: PLoS One. 2024 Mar 7;19(3):e0298601. doi: 10.1371/journal.pone.0298601 (PMC10919601; doi:10.1371/journal.pone.0298601)
Supplement: S1 Data — (ZIP) [file pone.0298601.s001.zip › Data/地级市Z_F工作报告.zip!/╔╜╢1⁄2/╚╒╒╒╩╨/└┤╫╘╣1⁄2╓┌║┼í╛┬φ┐╦╩2╛▌═°í┐_2007.docx]

各位代表：

现在，我代表市人民政府，向大会作政府工作报告，请予审议，并请市政协各位委员和其他列席会议的同志提出意见。

一、2006年政府工作回顾

2006年是实施日照市国民经济和社会发展第十一个五年规划的开局之年。一年来，在中共日照市委的坚强领导下，全市人民以邓小平理论和“三个代表”重要思想为指导，以科学发展观统领全局，深入贯彻党的十六大和十六届三中、四中、五中、六中全会精神，紧紧围绕“立足新起点、实现新跨越”的部署，努力促进经济可持续发展，积极构建社会主义和谐社会，全面完成了市十五届人大四次、五次会议确定的各项任务，实现了“十一五”的良好开局。

（一）国民经济保持快速健康发展，增长质量和效益明显提高。坚决贯彻国家宏观调控政策，坚持区别对待、有保有压，严把土地、信贷闸门和市场准入门槛，认真清理规范新开工项目，着力调整优化产业结构，全市经济迈上新的台阶。初步核算，2006年全市生产总值达到505.87亿元，比上年增长16.9%，增幅高于全省平均水平2.2个百分点；三次产业比例由上年的16.3：47.9：35.8调整为14.6：49.7：35.7。农业生产战胜严重干旱，实现增产增收。粮食总产达到105.2万吨，比上年增长4.8%；畜牧业、水产业产值分别增长6%和7.3%。工业生产快速增长，经济效益创历史新水平。规模以上工业实现增加值182亿元，比上年增长29.1%，增幅居全省第二位；实现利税、利润分别增长93.9%和118.2%，增幅均居全省第一位。全市规模以上工业企业达到727家，新增242家；主营业务收入过亿元的企业达到98家，新增22家，其中过10亿元的11家。高新技术产业产值比上年增长57.8%，占规模以上工业产值的比重上升2.1个百分点。民营经济增加值和营业收入分别增长28.8%和29.3%。城乡市场繁荣活跃，居民消费结构升级。市区新增大型超市、连锁店16家，营业额过亿元的商业零售企业达到5家。社会消费品零售总额实现145.1亿元，比上年增长16%。投资保持稳定增长，结构不断优化。规模以上固定资产投资完成223.9亿元，比上年增长20.1%。33项重点项目完成投资87.9亿元，制造业、新农村建设、社会事业等重点领域投资保持了较快增长。在经济快速发展的同时，节能降耗减排取得较好效果，万元GDP能耗、规模以上工业万元增加值取水量、COD排放总量等主要控制指标呈现下降趋势。

（二）社会主义新农村建设开局良好，县域经济发展加快。坚持以发展农村经济、促进农民增收为重点，大力调整优化农业结构，积极推进产业化、标准化、品牌化发展，农业综合生产能力进一步增强。全市经济作物面积比上年增长25%，市级以上农业龙头企业发展到45家，新创无公害和绿色食品品牌11个。坚持规划先行，编制完成了《日照市社会主义新农村建设总体规划》，在全省率先完成了新一轮小城镇总体规划修编，9处全国重点镇、省级中心镇和5处市级试点镇的规划建设顺利实施。“三农”投入力度持续加大，投资10多亿元加强了“路、水、电、医、学”等基础设施和社会事业建设，农村生产生活条件明显改善。认真落实各项支农惠农政策，全面取消农业税，直接减轻农民负担1918万元，落实农业综合开发、产业化经营和农村劳动力转移等专项扶持资金4000多万元，向农民发放粮食直补资金1889万元、成品油价格补贴7126万元、农机和良种补贴473万元。进一步简政放权，创新机制，优化服务，县域经济发展速度、质量、后劲同步提升，成为全市新的经济增长点。2006年县域生产总值、规模以上工业增加值、规模以上固定资产投资分别比上年增长17.8%、32.8%和30.5%，高于全市平均水平0.9、3.7和10.4个百分点。市高新技术产业园和莒县、五莲工业园被正式确认为省级开发区，县域经济发展空间进一步拓展。

（三）对外开放和招商引资深入推进，重点项目建设实现新突破。成功承办了第六次泛黄海中日韩经济技术交流会议，扩大了对外经济技术合作交流的渠道。坚持“对外主攻日韩，扩大港台，拓展欧美；对内接轨青岛、融入半岛，对接江浙沪粤”的招商工作策略，先后赴日本、韩国、美国、新加坡、香港和北京、上海、广东等地开展大规模的城市投资环境推介和招商引资活动，收到了良好效果。全年实际使用外商直接投资1.36亿美元，吸引内资310亿元。总投资4.9亿美元的韩国现代威亚汽车发动机项目获得国家发改委核准和商务部批准，成为山东省有史以来制造业规模最大的利用外资项目，标志着我市对外开放和工业化实现了新的突破。一期工程将于今年3月建成投产，有3个投资过千万美元的配套项目同步开工建设。亚太森博二期、华能日照电厂二期、岚山超超临界电厂等大项目取得了新的进展。进出口贸易快速发展，出口商品结构进一步优化。全年完成进出口总值39.15亿美元，比上年增长53.7%，其中出口增长38.9%，增幅均居全省第二位；工业制品出口比重提高了18.7个百分点。接轨青岛工作成效明显，去年两市之间先后组织四次大规模的经贸、科教考察交流活动，两市政府签署了进一步加强经济技术合作备忘录。全年我市共从青岛引进项目219个、到位资金19.1亿元，日照港至青岛港海运直通开航，青岛流亭国际机场在我市设立了虚拟空港。我市成功入选“中国投资环境百佳城市”，对外开放的环境进一步改善。

（四）港口发展实现亿吨跨越，特色经济迈出新步伐。日照港总体规划顺利通过交通部、省政府的审查，拉开了港口建设发展的新框架。港口基础设施建设全面推进，全年开工建设投资过千万元的项目22个，完成投资27亿元，新增吞吐能力2300万吨。特别令人振奋的是，经过开港开放20年的奋力拼搏，日照港跨入了亿吨大港行列，成为中国大陆沿海第九个、全省第二个亿吨大港。全年完成货物吞吐量1.1亿吨，比上年增长30.7%；集装箱运量25.27万标箱，增长18.3%。港口资本运营实现新突破，日照港股份有限公司在上海证券交易所成功上市，成为全省首家上市的港口企业，募集资金10.81亿元。港口经济快速发展，全年开工建设临港工业项目29个，完成投资45.4亿元；38家临港工业企业实现主营业务收入、利税、利润分别占全市工业总量的60%、67%和72%。口岸“大通关”建设取得新成效，港口发展环境进一步优化。日照至韩国平泽航线年客运量达到13.8万人次，比上年增长112.2%。我市作为全国首站城市，积极参与了“联合国新丝绸之路明珠城市”大型路演和“中国物流万里行·对话新大陆桥”等活动，扩大了东方桥头堡的对外影响。旅游经济快速发展，全年旅游人数达到1008.6万人次，实现旅游总收入50.8亿元，分别比上年增长28.9%和32.4%。“水上运动之都”建设迈出新步伐，成功承办了470级帆船世界锦标赛、全国帆船锦标赛总决赛等一系列重大赛事，首届中国水上运动会各项筹备组织工作扎实推进。大学科技园不断完善服务环境，在校大学生达到5.5万人。市政府与北京大学、山东海洋工程研究院、山东科技大学签订了全面合作协议，市校合作呈现出良好发展态势。

（五）深入开展生态市建设，城市规划建设管理水平不断提高。在广泛征求社会各界意见建议的基础上，完成了城市发展战略规划编制和城市总体规划修编工作，并提请市十五届人大五次会议审议批准，城市功能定位和空间布局进一步优化。编制完成了城市燃气、绿地系统、对外交通等专业规划和奥林匹克水上公园等重点项目规划，市区详细规划覆盖率达到91%。树立精品意识，高标准推进市政重点项目建设。新建城市基础设施项目19个、完成投资24.4亿元，城市载体服务功能进一步增强。奥林匹克水上公园建设完成投资4.5亿元，工程进度达到71%，今年6月将建成使用；投资5亿元、全长41公里的南北沿海公路建成通车，成为连接我市南北城区及港口的快速通道；北京路、济南路、临沂路等城市主干道贯通；日处理能力5万吨的第二污水处理厂建成运行，配套污水管网建设完成95%；新增城市绿化面积400万平方米、造林6万亩。深入开展城市环境综合治理，对城乡结合部和背街小巷实行重点整治，城市环境面貌明显改善。扎实推进生态市建设，对海域、岸线、耕地、森林、山体、河流等自然资源，实行严格的保护措施；积极发展循环经济，重视解决石材加工污染饮用水源等环境问题，依法整治违法排污企业，保护了全市生态环境。去年我市城市环境综合整治定量考核继续保持全省第一，城市空气质量在113个国家环保重点城市中名列前茅。我市先后获得国家级生态示范区、国家园林城市、国家环保模范城市、国家卫生城市等荣誉称号，通过了国家可持续发展实验区和国家节水型城市考核验收。

（六）财源建设成效明显，金融对经济社会发展的支持力度加大。适应转变政府职能、推行公共财政的要求，大力加强财源建设和综合治税、诚信纳税体系建设，制定出台了加强财源建设、税收征管、支出绩效考核、非税收入和专项资金管理等一系列政策措施，促进了财税收入的快速增长。全年地方财政收入达到20.76亿元，比上年增长42.5%，增幅居全省第二位；税收收入占地方财政收入的比重达到80.8%，居全省第三位。完成地方财政支出32.67亿元，比上年增长43.9%，基本保证了公共财政的需要。金融运行状况良好，年末金融机构各项存贷款余额达到420亿元和450亿元，分别比年初增加71亿元和104亿元；加上异地银行贷款余额66亿元，全市各项贷款余额突破500亿元。成功举办了2006日照银企合作促进会，签约贷款208亿元，已到位资金130亿元；与国家开发银行正式建立开发性金融合作关系，签约政策性贷款13.5亿元；组建成立金融服务中心和担保公司，搭起了银企合作的新平台。保险业服务领域不断拓宽，全年实现保费收入9.38亿元，比上年增长16.7%。

（七）和谐社会建设扎实推进，人民生活得到进一步改善。14项为民办实事重点项目全面完成。其中投资1.33亿元，建成925个村的自来水工程，新增受益人口63万人；新型农村合作医疗农民参合率达到92.1%；投资859万元，对全市10处重点乡镇卫生院进行了整修改造；新建农村公路460公里，硬化路通村率提高到98%；投资3422万元，改造农村中小学危房4.33万平方米；对24000名农村贫困家庭学生落实了“两免一补”政策；农村“五保”对象集中供养率达到35%，比上年末提高15个百分点；对625户城市中低收入家庭实施了经济适用住房货币直补，对291户市区低收入家庭落实了廉租房补贴政策；“万村千乡”市场建设工程进展顺利，新建农村社区综合服务中心、农家店663处。就业和社会保障工作进一步加强。全年新增城镇就业再就业4.14万人，城镇登记失业率控制在3.2%，新增农村劳动力有组织跨区域转移2.63万人。实施农村居民最低生活保障制度，城市低保实现动态管理下的应保尽保，全年发放保障金1095万元。新增企业基本养老保险参保职工8250人，农村社会养老保险参保农民达到61.3万人。各项社会事业取得新进步。成功举办了海洋科技论坛暨科技合作洽谈会，聘请在青院士、专家成立了海洋科技专家咨询委员会。新建市级以上企业技术中心和工程技术研究中心25家，争创中国名牌2个、山东名牌17个。各类教育协调发展，小学、初中入学率分别达到100%和99.8%，高中段教育普及率达到87%，职业教育、成人教育年培训60万人次。城市社区卫生服务和传染病防控工作得到加强，市“120”紧急救援指挥中心组建启动，公共卫生服务水平有了新的提高。加大市场监管力度，完善农村食品药品监管网络和供应网络，保证了群众饮食及用药安全。文化事业不断发展，两城镇等3处遗址被列为全国重点文物保护单位。土地、海洋、森林资源保护工作进一步加强，耕地总量保持动态平衡。人口自然增长率控制在4.2‰，完成了省委、省政府下达的人口控制指标。国防后备力量建设与经济社会建设协调发展，优抚安置政策进一步落实，双拥共建工作取得新成效。新闻出版、广播电视、民族宗教、外事侨务、妇女儿童、老龄、残疾人、档案、史志、人防、地震、气象、驻外机构、红十字会等工作也都取得了新的成绩。民主法制和“平安日照”建设深入推进。“五五”普法规划顺利实施，基层民主政治建设不断加强，社区建设力度进一步加大。认真做好信访工作，高度重视安全生产，大力加强社会治安综合治理，“平安日照”建设成效明显。全年刑事案件发案、安全生产事故起数分别比上年下降2.3%和10.6%。人民生活不断改善，收入水平进一步提高。去年城市居民人均可支配收入达到11040元，比上年增长12.6%；农民人均纯收入达到4645元，增长12.7%。

（八）政府自身建设得到加强，社会管理和公共服务水平进一步提高。市政府认真执行市人大及其常委会的决议、决定，自觉接受市人大及其常委会的依法监督和市政协的民主监督。全年办理人大代表建议38件、政协委员提案154件，办复率、基本满意率均达到100%。深入实施政府工作“阳光工程”，全面落实政府信息公开、新闻发布会、重大社会公共事项决策听证等制度，增强了政府工作的透明度。完善市长接听日制度，做好市长公开电话、市长信箱、“行风在线”直播等方面的工作，民意渠道进一步畅通，解决了一批事关群众切身利益和社会关注的问题。加强电子政务建设，日照政务网荣获“中国电子政务最佳运维服务机构20强”。积极推行充分授权、市区联合审批等制度，行政服务效能有了新的提高。着力推进公共应急管理机制建设，制订出台应急预案30个，成立了省级危险化学品港口应急救援中心，举行突发公共卫生事件、森林火灾扑救和重大动物疫病防控等大型应急处置演练16次。认真学习贯彻《行政许可法》、《公务员法》和《监督法》，大力推行行政执法责任制和过错追究制，依法行政工作得到加强。落实党风廉政建设责任制，加强行风建设，强化行政监察和审计监督，深入开展商业贿赂专项治理，从严查处违法违纪案件，全年共处分违反政纪人员26人，形成了勤政廉洁的政务环境。

各位代表，过去的一年，我们经历考验，克服困难，进一步巩固和发展了历届市领导班子开创的良好局面，迈出了“十一五”发展坚实的一步。成绩来之不易，这是省委、省政府亲切关怀，市委正确领导的结果，是人大、政协和社会各界监督支持的结果，是全市人民解放思想、开拓创新、顽强拼搏、真抓实干的结果。在此，我代表市人民政府，向工作在全市各行各业的广大劳动者和建设者，向给予我们支持监督的各级人大代表、政协委员，向各民主党派、工商联、无党派人士、各人民团体，向离退休老领导、老同志，向驻日照人民解放军和武警官兵，向所有关心支持日照建设的社会各界人士和海内外朋友，表示崇高的敬意和衷心的感谢！

在肯定成绩的同时，我们也清醒地认识到存在的差距和问题：经济结构性矛盾比较突出，一产比重仍高于全省平均水平，高新技术产业总量小，服务业发展水平有待提高，产业结构优化升级的任务很重；经济外向度较低，利用外资规模小；县域经济比较薄弱，农村二、三产业发展滞后，县乡财政仍比较困难；经济增长方式比较粗放，资源利用水平偏低，节能降耗、环境保护和集约节约用地压力大；影响社会和谐的因素仍然存在，部分群众在生活、就业、就医、社保、住房等方面还存在困难；政府职能和作风转变不够到位，一些部门和单位服务意识不强、办事效率不高，经济发展环境还需进一步改善。对这些问题，市政府将高度重视，采取有力措施，认真加以解决。

二、2007年政府工作的主要任务

2007年是我市落实科学发展观、构建和谐社会的重要一年，也是加快实施“十一五”规划，立足新起点、实现新跨越的关键一年。综合分析形势，我们既具备了加快发展的机遇条件，也面临着新的考验。国际资本和产业转移步伐进一步加快，国家宏观调控政策保持连续性和稳定性，省委、省政府加强半岛城市群和鲁南经济带开发建设，一批事关日照长远发展大项目的相继建成投产，都为我市加快发展带来了新的机遇和动力；WTO过渡期结束、税收政策调整、各项改革深化、财政刚性支出增多、生产要素制约趋紧、资源环境压力加大等，也给我市加快发展、扩大开放和构建和谐社会带来新的考验。全市各级政府及政府组成人员要认清形势，用心把握，进一步提高贯彻落实科学发展观的自觉性和坚定性，扎扎实实地做好今年的各项工作。

今年政府工作总的要求是：坚持以邓小平理论和“三个代表”重要思想为指导，以科学发展观统领经济社会发展全局，按照省委、省政府和市委的总体部署，突出又好又快发展这一主题，牢牢把握调整结构、转变方式、改革开放、促进和谐四个关键环节，深入实施港口立市、工业强市、科教兴市、生态建市“四大战略”，积极推进招商引资、临港产业、县域经济、高新技术、现代农业、服务业、生态市建设、公共服务和改善民生，努力开创我市经济社会科学发展、跨越发展的新局面。在工作中，要注重落实宏观调控政策、提高经济发展的质量和效益，注重节能降耗、自主创新、环境保护和集约节约用地，注重以人为本、改善民生，注重狠抓落实、务求实效。今年我市经济社会发展的主要预期目标是：生产总值比上年增长15%，地方财政收入增长16%，规模以上固定资产投资增长20%，社会消费品零售总额增长15%，外贸进出口总值增长17%，实际利用外商直接投资增长25%，城市居民人均可支配收入增长9%，农民人均纯收入增长8%，新增城镇就业3.85万人，登记失业率控制在3.5%以内，居民消费价格总水平涨幅控制在3%以内。主要约束目标为：万元GDP能耗降低4.5%，二氧化硫排放减少9.7%，COD排放降低4.4%，人口出生率和自然增长率控制在13.2‰和6.6‰以内。围绕上述总体要求和目标，今年重点抓好以下八项工作：

（一）扎实推进社会主义新农村建设，全面繁荣农村经济。坚持把“三农”工作作为全部工作的重中之重，认真贯彻今年中央1号文件精神，围绕提高农业综合生产能力和增加农民收入，组织实施好我市新农村建设《总体规划》。要因地制宜，坚决防止“一刀切”和违背群众意愿的形象工程，确保我市新农村建设取得实效。

一是大力发展现代农业。按照用现代产业体系提升农业、用现代物质条件装备农业、用现代科学技术改造农业、用现代经营形式推进农业、用培养新型农民发展农业的要求，加快传统农业向现代农业转变。在稳定粮食生产的基础上，进一步调整优化农业结构，重点发展茶、桑、果、菜等特色高效农业，年内新增经济作物面积5万亩。大力发展食草、节粮型畜禽，实施无公害水产品行动计划，推广规模养殖、生态养殖，畜牧业和水产业产值分别增长6%和10%。加大对45家市级农业龙头企业扶持力度，积极发展农民专业合作组织，提高农业产业化经营水平。大力推进农业科技进步，加强农业水利化、机械化、信息化建设，建立健全农产品标准、质量检测监管和动植物疫病防治体系，启动市区农产品监测中心建设。抓好新型农民科技培训和职业技能培训，鼓励和引导农村剩余劳动力向二、三产业转移，努力增加农民的非农就业和非农收入，全年扶持培训农村劳动力1万人，有组织转移2万人以上。

二是加强农村基础设施和社会事业建设。加快推进“北水南调”二期、沭河综合治理一期和第二批百座水库除险加固等重点工程，抓好节水灌溉、小流域综合治理等农田水利基本建设，进一步提高农业抗御自然灾害的能力。集中力量抓好中心镇、中心村的试点工作，以点带面稳步推进新农村建设。加强村庄规划和村居环境整治，继续推进“路水电气医学”等农民最急需的生产生活基础设施建设。年内完成农村公路改造300公里，新建12个农村客运站，让农民群众出行更加便捷。投资4860万元，新建280个村的自来水工程，全市农村自来水普及率达到80%，让农民群众饮水更加安全。搞好农村改水、改厕、改灶、改圈，年内新增沼气用户2250户。投入7163万元，对农村义务教育阶段学生实施“两免一补”，全部免除农村24万名学生杂费，对16000名贫困家庭学生免费提供课本并补助寄宿生生活费。投入2145万元，改善农村“五保”对象集中居住条件，“五保”对象集中供养率提高到45%。完成13处乡镇卫生院改造建设，各级财政对新型农村合作医疗人均补助额提高到40元。加快农村现代流通体系和食品药品监管供应网络建设，新建农村社区综合服务中心8处、综合超市40处、农家店300处。通过努力办实事，让广大农民得到更多实惠。

三是加快提升县域经济综合实力。以壮大县乡财力、增加农民收入为目标，以工业为主导、开放为动力、农业为基础、城镇和园区为支撑，加快发展县域经济。充分发挥港口、资源、产业等优势，积极引进临港工业配套型、产业协作型、农产品加工带动型、财税增收型项目落户县域，培育壮大一批对县乡财力具有支撑作用的骨干企业，全年县域规模以上工业增加值和固定资产投资确保增长20%和30%以上。进一步简政放权，优化环境，强化扶持，加快发展各具特色的镇域经济和农村集体经济，积极培育经济强镇、经济强村，努力形成市、县、镇、村经济协调发展的良好局面。

四是深入推进农村综合改革。稳定完善农村土地承包经营制度，认真落实各项支农惠农政策，建立健全防止农民负担反弹的长效机制，切实维护农民权益。加快推进以乡镇机构、农村义务教育和县乡财政管理体制为主要内容的综合改革，不断增强农村发展活力。努力化解乡村债务，坚决防止新债发生。加强乡村基层组织建设，健全完善村民议事、村务公开等制度，提高农村民主管理水平。今年各级财政支农资金的增量要高于上年，固定资产投资用于农村的增量要高于上年，土地出让收入用于农村建设的增量要高于上年。各级都要站在统筹城乡发展、全面建设小康社会的大局，确保对“三农”投入的到位，确保各项支农措施的落实。

（二）全方位扩大对外开放，大力开展招商引资。对外开放，是我市发展的最大优势，也是潜力所在。要进一步解放思想，转换机制，抓住国际国内资本、产业、技术、人才战略转移的机遇，举全市之力抓好对外开放和招商引资。

一是切实加大项目推进和落实力度。坚持既定的招商策略不动摇，主动走出去、请进来，扩大外引内联，年内组织赴欧美、韩、日、台等地区开展重点招商引资活动。进一步创新招商方式，加强专业招商、产业招商和以商招商，努力形成大招商、招大商的格局。实行重点项目责任制，对当前272个重点招商项目和今年新招引的项目，落实专人分工推进，实施全程跟踪调度，提高项目成功率和外资到位率。全年确保实际利用外商直接投资增长25%、利用内资增长30%以上。

二是切实提高招商引资的质量与水平。认真把握国家利用外资的政策导向，围绕发挥我市优势、优化产业结构和加强财源建设，重点推进港口建设、临港工业、先进制造业、高新技术和环保产业、现代服务业、城市基础设施等领域的招商引资，努力提高利用外资的质量。紧紧依托现代威亚汽车发动机项目，抓好配套项目招引和产业集群建设，加快韩资项目向我市集聚，使日照真正成为承接韩日产业转移的重点地区。把推进企业上市作为招商引资的新方式，鼓励扶持优质企业到境内外上市，积极利用资本市场促进我市经济发展。

三是切实优化投资环境。加强港口、公路、铁路等基础设施建设，进一步改善对外开放和招商引资的环境。年内完成港口投资26亿元以上，抓好西港区二期、矿石码头二期等13项重点工程。开工新菏兖日铁路电气化改造工程，做好日（照）黄（岛）铁路、日（照）连（云港）盐（城）铁路和日照至潍坊、岚山至临沂高速公路建设准备工作。加快推进电子口岸建设，开工建设国际贸易与航运服务中心，完善港口集疏运体系，进一步提高通关效率。抓好省级开发区等重点园区的规划建设，不断增强承载能力和服务功能。下大力抓好软环境建设，积极开展作风建设年活动，切实解决办事效率低、服务意识弱、服务质量差等问题，努力形成亲商、安商、扶商、富商的浓厚氛围。

四是切实转变外贸增长方式。加快调整出口产品结构，支持加工贸易和机电产品出口，引导出口企业从数量型、创汇型向质量型、效益型转变。全年加工贸易出口增长10%，工业制品出口增长50%。抓住国家调整进口关税等有利时机，扩大先进技术、重大装备、关键零部件及重要资源、原材料进口。积极发展对外经济技术合作，鼓励有条件的企业到国外投资办厂、开展工程承包和劳务输出。

（三）加大经济结构调整力度，推动产业优化升级。认真贯彻落实国家宏观调控政策，坚持走新型工业化道路，抓住结构调整这条主线，突出发展先进制造业、高新技术产业和现代服务业，加快提升产业素质和竞争力。

一是培植壮大临港工业。坚持“大项目—产业链—产业基地”的发展方向，大力培植钢铁、浆纸、粮油加工、液体化工、木制品加工、机械制造、造船等临港产业，加快发展汽车及配件、食品加工、纺织服装、新型建材等优势产业，积极争上投资规模大、科技含量高、带动能力强的临港工业项目，支持50户重点企业发展和百项重点工业项目建设，形成新的经济增长点。依托钢铁、发动机等骨干项目，培育关联配套产业，促进企业集聚和产业集群发展，加快构建具有日照特色和竞争优势的先进制造业基地。

二是大力发展高新技术产业。重点培植电子信息、生物技术、精细化工、新材料及海洋新兴产业，全年高新技术产业产值突破100亿元，占规模以上工业产值的比重提高2个百分点以上。推进高新技术产业园建设和省级开发区产业升级，大力引进国内外知名高科技企业和项目。充分利用先进技术、信息化技术，改造提升传统产业和重点企业的工艺、装备和管理水平，年内完成技改投资96亿元。

三是繁荣发展现代服务业。发挥我市港口、区位、生态环境等优势，大力发展交通运输、现代物流、金融、信息、中介等生产性服务业，积极培育商贸、旅游、餐饮、房地产、文化等消费性服务业，努力提高服务业规模、质量和水平，全年服务业增加值突破200亿元。完善港口物流功能，加强港航、港企、港港合作，增开国际国内航线，积极开拓腹地市场，扩大为鲁南经济带、新亚欧大陆桥经济带服务的范围，不断增强港口核心竞争力。全年港口货物吞吐量达到1.26亿吨以上，集装箱运量突破50万标箱。坚持旅游开发与经济社会发展全方位结合，进一步整合优化旅游资源，完善旅游环境，提高服务质量和档次，努力把旅游业打造成我市国民经济的主导产业。全年旅游人数、旅游收入分别增长18%和20%。

四是促进区域经济协调发展。坚持优势互补、共同发展，着力提升各区县经济发展水平，促进市与县区、县区与县区之间经济协调健康发展。立足市域产业体系、资源能源供给和生态环境现状，统筹规划，合理布局，推动更多的外向型项目、临港工业项目向区县延伸和集聚，逐步形成分工明确、特色鲜明、良性互动的产业体系。加强区域经济合作特别是与半岛城市群、鲁南经济带、新亚欧大陆桥经济带、泛黄海经济圈的合作，做好接轨青岛的工作，实现交通、旅游、文化、市场资源共享和人才、技术、资金等要素无障碍流动，促进区域经济、城乡经济一体化发展。

五是大力发展民营经济。认真落实国家和省、市关于鼓励支持民营经济发展的一系列政策措施，进一步放宽市场准入，简化审批程序，放手发展民营经济，推动我市民营经济上规模、上水平。力争民营经济增加值占全市生产总值的比重比上年提高4个百分点。加强中小企业服务体系建设，加快实施中小企业成长计划，培育一批拥有核心技术、竞争力强的民营龙头企业和具有创新意识、高度社会责任感的民营企业家。

（四）突出抓好自主创新、节能降耗、环境保护和集约节约用地，加快转变经济增长方式。认真落实节能降耗、环境保护和土地管理责任制，严格考核机制和问责制，把经济社会发展切实转入可持续发展轨道。

一是围绕建设创新型城市，努力提高自主创新能力。突出企业技术创新、农业科技创新和海洋科技创新三个重点，积极推进企业研发中心、科技孵化器和科技创新平台建设，加快构建以企业为主体、市场为导向、政产学研金相结合的科技创新体系。年内创建10个市级以上工程技术研究中心，取得重要科技成果60项以上，专利申请量完成700件以上。积极实施品牌战略，力争年内新增中国名牌产品1个、山东名牌产品8个。进一步完善大学科技园载体服务功能，吸引高科技研发机构入园，加快科技成果孵化基地建设，鼓励高校与我市开展科技创新合作。认真落实人才强国战略，加强科技创新人才队伍建设，为经济社会发展提供强有力的人才支撑。

二是围绕建设节约型社会，切实抓好节能降耗减排节水节地等工作。着力抓好钢铁、电力、造纸、化工、建材等重点行业和50家重点耗能企业的节能降耗减排，年内规模以上工业企业万元增加值能耗降低4.5%、取水量降低7%。严把工业项目节能门槛，坚决淘汰浪费能源、污染严重的企业和落后工艺、技术、产品。大力发展节能、节材、节水型建筑和基础设施，积极推广利用沼气、太阳能、风能、醇醚燃料等清洁能源，提高社会能源利用效率。加快土地利用总体规划修编，严格执行国家土地政策，依法保护耕地特别是基本农田。搞好存量土地挖潜利用，落实投资强度、建设用地容积率和建筑密度等控制性指标，大力发展节地型经济、节地型工业、节地型项目。深化水资源管理体制改革，严格执行工业生产用水定额标准，发展节水型农业，推广中水回用，巩固提高“国家节水型城市”创建成果。

三是围绕建设环境友好型社会，扎实推进生态市建设。“蓝天、碧海、金沙滩”是日照得天独厚的环境优势，也是不可再生的宝贵资源。各级政府要把环境优先的观念贯穿于经济社会发展的全过程，严格执行“环保第一审批”和“一票否决”制度，切实搞好生态环境建设。要全面实施农村小康环保行动计划，抓好农业面源污染和土壤污染防治，加快推进环境优美乡镇、文明生态村建设。加强对日照水库等饮用水源地保护，采取有力措施整治石材加工污染问题。抓好沭河、洪凝河、崮河、傅疃河、绣针河等流域综合治理，确保主要河流水质达到水环境功能区要求。加大对重点污染源的治理力度，严格控制二氧化硫等空气污染物排放，确保城市空气质量继续保持优良水平，年内日照钢铁厂、华能日照电厂脱硫项目建成并投入运行。认真执行《日照市循环经济促进办法》，抓好第二批10家企业的循环经济试点工作，建好生态工业示范园区和生活垃圾分类回收示范社区，积极推进循环型社会建设。要严格落实对山体、绿地、滨海湿地、森林、岸线及海域等生态环境资源的保护措施，做到精心规划、精心建设、精心管理，坚决杜绝盲目开发带来的环境破坏，全力保护好日照的青山绿水、碧海蓝天。

（五）以迎办水运会为契机，加快推进国际水准滨海城市建设。今年8月28日首届中国水上运动会将在我市举办，这是我市打造水上运动之都、加快城市建设的大好机遇。我们要抓住用好这一机遇，全面提升城市规划建设管理水平。

一是努力提升城市规划设计水平。按照市十五届人大五次会议审议批准的城市发展战略规划和新一轮城市总体规划，扎实做好控规、详规、专业规划和城市设计工作，高标准完成市区北部生态旅游区控规、滨海岸线详规，加快编制海域使用规划、供热专业规划、城市对外交通规划和涛雒地区规划，力争年内市区详细规划覆盖率达到100%。严格按照规划规范项目建设，严厉查处违法建设，切实维护规划的法定性。加强对区县和小城镇规划修编的指导服务，实现主城区与县城及小城镇统筹发展。

二是努力提升城市建设品位。坚持以国际水准建设滨海城市，强化精品意识，注重城市品位，体现城市特色，高标准、高质量地推进城市建设，年内开工建设21项市政重点工程。切实抓好奥林匹克水上公园建设，确保建成一流的比赛场地、旅游景区和休闲健身公园。搞好聊城路、滨州路等城市道路建设，完成沙墩河整治工程，加快污水处理主干管网及垃圾处理厂二期工程建设，开工建设新市区客运站，着力构筑城市大交通、大生态体系，进一步增强城市载体服务功能。

三是努力提升城市综合环境质量。投资1.42亿元，启动市区居住环境综合整治工程，贯通7条对居民出行影响较大的城市次干道，进一步抓好城乡结合部、旅游景点、居民小区、农贸市场、背街小巷、建筑工地等重点部位的环境整治，巩固提高边界环境整治成果。坚持统一规划、综合开发、先行试点、逐步推开，年内启动东港区湖西头村和万平社区两个城中村居的改造试点工作。搞好滨海地带、沿海公路绿化美化和城市道路绿化工程，年内新增城区绿化面积500万平方米、造林5万亩。加强城市综合执法和文明执法，巩固长效管理机制，加大交通秩序、市场秩序治理力度，努力营造繁荣有序、整洁卫生、优美舒适的市容环境和人居环境，争创“中国人居环境奖”。

（六）坚持以人为本，切实解决人民群众最关心、最直接、最现实的利益问题。始终把扩大就业放在经济社会发展的突出位置。实行积极的就业政策，落实好促进就业再就业的各项政策措施。认真组织实施技能扶贫工程，免费开展再就业培训6800人。做好城镇新增劳动力、被征地农民、高校毕业生和复员转业军人就业工作，鼓励支持自主创业、自谋职业。积极开发公益性就业岗位，重点帮助零就业家庭和困难群体就业。严格实施农民工工资支付保证金制度，坚决查处拖欠工资行为。

健全完善社会保障和社会救助体系。进一步扩大社会保障覆盖面，完善企业职工基本养老保险制度，提高农民养老保险参保率，逐步建立覆盖城乡居民的社会保障体系。年内劳动合同签订率提高到91%，社会保险单位参保覆盖率提高到90%。加强社保基金监管，确保基金安全和保值增值。完善失业保险政策和城镇职工基本医疗保险制度，统筹解决灵活就业人员、进城务工人员等社会群体的社会保障问题。建立被征地农民基本养老保险制度，年内在东港区、日照经济开发区启动试点工作。全面实施农村居民最低生活保障制度，低保标准每人每年不低于700元。大力发展社会救助、社会福利和慈善事业，切实解决特困群众就医难、上学难、生活难等问题。建立政府、社会多渠道筹资机制，对患大病的困难群众进行救助。

继续实施经济适用住房货币直补和困难家庭廉租房租金补贴制度，出台城中村改造政策办法，进一步调控房地产市场，加强多层次住房供应体系建设，满足群众住房需求。强化食品药品监督管理，确保人民群众饮食及用药安全放心。深入整顿和规范市场经济秩序，严厉打击哄抬物价、欺行霸市、制假售假等行为，营造质优价廉、放心安全的消费环境。扎实做好大中型水库移民后期扶持各项工作，确保移民政策落到实处。高度重视并认真解决土地征用、房屋拆迁、企业改制、供气供热、公交、医疗、教育、物价等方面损害群众利益的突出问题。今年，市委、市政府按照落实科学发展观、构建社会主义和谐社会的要求，确定了村村通自来水、“五助”惠残等12项为民办实事重点项目。各级政府和各责任单位要高度重视，集中人力物力财力，认真履行承诺，确保把每件实事办实办好，把党和政府的温暖送到群众特别是困难群众当中。

（七）统筹发展各项事业，扎实推进和谐社会建设。优先发展教育，完善农村义务教育经费保障机制，高水平、高标准普及九年制义务教育。加快市区中小学校布局调整，确保市实验学校、泰安路小学按期投入使用。提高高中段教育普及水平，大力发展职业教育和成人教育，搞好高等技工学校建设。加强基层教师队伍建设，着力解决农村中小学教师数量不足、年龄偏大等问题，积极推进素质教育。突出公共卫生、农村卫生和城市社区卫生三个重点，努力为群众提供安全、有效、方便、价廉的公共卫生和基本医疗服务。年内市区新建5处社区卫生服务中心和30处社区卫生服务站，社区卫生服务人口覆盖率达到60%以上。广泛开展群众性的文化活动，培育发展文化事业和文化产业，加强对外文化交流，加快城乡文化设施建设。加快推进村村通有线电视工程，扩大有线电视和数字电视网络覆盖面。继续稳定低生育水平，不断提高人口素质，统筹解决人口问题。积极开展全民健身活动，全力承办好首届中国水上运动会，努力办成一届“成功、精彩、难忘”的体育盛会，并促进旅游、商贸、通信、文化等相关产业的发展。大力支持发展残疾人事业，维护好残疾人的合法权益。同时，积极发展新闻出版、老龄、妇女儿童、外事、侨务、对台、档案、史志、气象、防震救灾、人防等各项事业。

加强民主法制和精神文明建设。深入开展“五五”普法教育，全面推进依法治市。强化基层民主政治建设，做好村委会换届选举工作。认真落实民族宗教政策，依法管理宗教事务。大力加强思想道德建设，深入开展社会主义荣辱观教育活动，推进和谐社区、和谐村镇等群众性精神文明创建活动。以“迎水运会、创文明城”为主题，广泛开展系列文明创建活动，全面提高市民素质和社会文明程度，努力争创省级文明城市。加强全民国防教育和国防后备力量建设，深入开展双拥共建活动，力争国家双拥模范城“三连冠”。

扎实推进“平安日照”建设。坚持重心下移，一线布警，加强城乡治安防控体系建设，严厉打击各类违法犯罪活动。认真抓好安全生产，严防各类重大事故发生，确保完成省政府下达我市的控制指标。高度重视做好人民来信来访工作，坚决防止和纠正损害群众利益的行为。完善突发公共事件应急管理机制，抓好预案演练，提高快速反应和综合控制能力，努力为经济社会发展创造安全、稳定、和谐的社会环境。

（八）加强政府自身建设，提高政府工作人员科学发展和服务社会的能力。各级政府及其组成部门要按照“为民、务实、清廉”的要求，切实转变作风，加快推进管理创新，努力建设法治政府、服务政府、责任政府和效能政府。

一是加强学习，切实提高公务员素质。在政府系统大力倡导勤奋好学、学以致用的良好风气，努力建设学习型政府、学习型机关。广大公务员要认真学习政治理论，提高理论素养，不断加强工作中的理论指导；努力学习现代经济、科技、社会管理和法律知识，树立世界眼光和战略思维，提高贯彻落实科学发展观、构建和谐社会、驾驭市场经济的能力；加强政府工作人员宗旨观念教育，牢固树立执政为民的理念，切实增强为人民群众服务的本领，多办顺应民意、化解民忧、为民谋利的实事。

二是切实转变政府职能，提高社会管理和公共服务能力。各级政府在充分履行经济调节和市场监管职能，推进经济又好又快发展的同时，要注重社会管理和公共服务，把工作的着力点放在开展平安建设、发展社会事业、打造人居环境等关系人民群众切身利益的民生问题上来，加强政府社会管理和公共服务的绩效考核。分类推进事业单位改革，创新事业单位运行机制。要加快建立完善公共财政体制，深化部门预算、国库集中支付、支出绩效评价、政府采购和国有资产管理等改革，把财力、物力、人力更多地向社会管理和公共服务领域倾斜，努力让广大群众特别是困难群众分享到改革发展的成果。

三是加强行政效能建设，增强政府的执行力和公信力。认真贯彻落实中央和省、市委的决策部署，紧密结合当地实际，创造性地开展工作。完善重大事项集体决策、专家咨询、社会公示与听证相结合的决策机制，切实提高决策科学化、民主化水平。实行政务公开，把人民群众最关心的事项作为公开的重点，增强政府工作透明度。发挥好市长公开电话、市长信箱的作用，进一步畅通民意渠道。完善行政审批大厅集中服务，加快市区网上并联审批改革，规范各部门窗口式、一站式服务。大力推进电子政务建设，推行服务承诺、首问负责、一次告知、限时办结等工作制度，认真落实行政问责和责任追究，全面提高行政效率和服务质量。

四是坚持依法行政，促进廉政建设。认真贯彻落实《行政许可法》、《公务员法》、《监督法》，积极组织实施依法行政第四个五年规划，严格依法行使权力、履行职责、接受监督。坚持向人大及其常委会报告工作和向人民政协通报情况的制度，市政府常务会议邀请市人大、政协领导同志参加，自觉接受人大及其常委会和人民政协的监督。重视做好人大代表议案、建议、批评、意见和政协委员提案的办理和落实工作，广泛听取民主党派、工商联、无党派人士和各人民团体的建议意见。认真贯彻中纪委七次全会精神和胡锦涛总书记倡导的八个方面良好风气的要求，全面落实党风廉政建设责任制，健全教育、制度、监督并重的惩治和预防腐败体系。强化对公共权力运行的制约和监督，在国有资产转让、房地产开发、政府采购、招投标等重点领域，建立起严格的监督制度和权力制约机制。深入开展商业贿赂专项治理，坚决纠正损害群众利益的不正之风，严厉查处各类违法违纪案件。牢记“两个务必”，大力加强政风建设，坚持求真务实，艰苦奋斗，勤俭办一切事业。

各位代表，回顾过去的一年，全市人民用勤劳和智慧创造了辉煌的业绩；展望2007年，站在新的起点，面向日照发展的美好未来，我们既满怀信心，又深感责任重大。让我们紧密团结在以胡锦涛同志为总书记的党中央周围，在中共日照市委的坚强领导下，同心同德，开拓进取，奋力拼搏，为建设美丽富强、文明开放的新日照而不懈努力，以优异成绩迎接党的十七大和省、市党代会的胜利召开！
